# Beta-catenin inhibits TR4-mediated lipid accumulation in 3T3-L1 adipocytes via induction of Slug

**DOI:** 10.1186/s13578-020-00482-4

**Published:** 2020-10-15

**Authors:** Hojung Choi, Sung-Soo Park, Seung-Jin Kim, Eungseok Kim

**Affiliations:** 1 Deptartment of Biological Sciences College of Natural Sciences, Chonnam National University, 77 Yong-bong-ro, Buk-Gu, Gwangju, 61186 Republic of Korea; 2 College of Pharmacy and Gachon Institute of Pharmaceutical Science, Gachon University, 191 Hambakmoe-ro, Yeonsu-gu, Incheon, 21936 Republic of Korea; 3 Research and Development Division, World Institute of Kimchi, 86 kimchi-ro, Nam-gu, Gwangju, 61755 Republic of Korea; 4 Department of Biochemistry, College of Natural Sciences, and Kangwon Institute of Inclusive Technology, Kangwon National University, Chuncheon, 24341 Republic of Korea

**Keywords:** TR4, β-catenin, Slug, 3T3-L1 adipocytes

## Abstract

**Background:**

TR4, an orphan nuclear receptor plays a key role in glucose and lipid metabolism by regulating the expression of genes involved in energy metabolism. We previously reported that overexpression of TR4 in 3T3-L1 adipocytes promotes lipid accumulation in part by facilitating fatty acid uptake and synthesis, indicating that TR4 tightly regulates lipid homeostasis during adipogenesis. Here, we report that β-catenin suppresses TR4 transcriptional activity and that this inhibition is achieved through induction of Slug gene, a well-known transcription repressor in a variety of cells

**Methods:**

To generate the stable cell line, 3T3-L1 cells were transfected with plasmids then cultured in presence of geneticin and/or blasticidin for 2 weeks. The lipid accumulation was measured by Oil Red O. The TR4-Slug and TR4-β-catenin interactions were checked by GST pull-down and mammalian two-hybrid assay. The TR4 transcriptional activities on various promoters were measured by luciferase activity. To check the binding affinity of TR4, we performed the gel shift and chromatin immunoprecipitation (ChIP) assay. Gene expression was detected by RT-qPCR at the mRNA level and western blotting at the protein level.

**Results:**

Stable overexpression of Slug gene in 3T3-L1 preadipocytes strongly inhibited differentiation of 3T3-L1 preadipocytes. Using GST pull-down, gel shift and ChIP assays, we found that Slug abolished the formation of TR4 homodimers through direct interaction with TR4 and reduced the binding affinity of TR4 for its response elements located in TR4 target gene promoters such as fatty acid transport protein 1 and pyruvate carboxylase. Consistently, Slug inhibited TR4 target gene expression and was accompanied by repression of TR4-induced lipid accumulation in 3T3-L1 adipocytes.

**Conclusions:**

Our results demonstrated that Slug inhibits 3T3-L1 adipogenesis through suppression of TR4 transcriptional activity.

## Introduction

TR4, an orphan nuclear receptor, shows circadian expression in key metabolic tissues, such as the liver, adipose, and muscle, and plays a key role in glucose and lipid metabolism by regulating the expression of genes involved in energy metabolism [1–3]. Recent studies from our group and others have reported that systemic loss of TR4 in mice leads to reduced body weight gain and triglyceride accumulation in adipose tissue and the liver and increased insulin sensitivity [4, 5]. In contrast, overexpression of TR4 in 3T3-L1 adipocytes promotes lipid accumulation in part by facilitating fatty acid uptake and synthesis, indicating that TR4 tightly regulates lipid homeostasis during adipogenesis [1, 2].

Adipocytes are the principal cells for lipid storage and mobilization in response to nutritional demands. The molecular mechanisms underlying adipocyte differentiation have been extensively studied using well characterized in vitro model systems such as 3T3-L1 preadipocyte cells [6]. Adipogenic stimuli initially induce the expression of C/EBPβ and C/EBPδ and then stimulate the expression of C/EBPα and PPARγ, which cooperatively promote the adipogenic program by inducing lipogenic genes, leading to the formation of fat-accumulated mature adipocytes [7, 8]. In addition, other transcription factors, including TR4, also control adipogenic gene expression during adipocyte differentiation [8, 9]. Recent studies have shown that activation of Wnt/β-catenin signaling in 3T3-L1 cells induced by Wnt overexpression or a constitutively active β-catenin mutant that lacks the GSK-3β phosphorylation site inhibits adipogenesis [10]. Stimulation of Wnt/β-catenin signaling suppressed the induction of C/EBPα and PPARγ expression, and consequently, inhibited adipogenesis [11, 12].

Snail is a member of a family of zinc-finger transcription factors that play crucial roles in various cellular programs such as mesoderm formation and epithelial-mesenchymal transition [13]. In vertebrates, Snail has been reported as a negative regulator of adipogenesis and a mediator of the Wnt signaling pathway [14, 15]. Interestingly, previous studies showed that Slug (SNAI2), another member of the Snail family, is also induced by β-catenin signaling [16, 17]. Compared to other key adipogenic transcription factors that are induced during adipocytic differentiation of 3T3-L1 cells, TR4 levels remain relatively unchanged [18]. Thus, we hypothesized that a negative regulator of adipogenesis may inhibit TR4 function in preadipocytes; thus, TR4 adipogenic function may be induced by a decrease in a negative regulatory signal, such as Wnt/β-catenin, as adipocyte differentiation progresses. In this study, we demonstrated that β-catenin acts as a negative regulator of TR4 activity. Overexpression of β-catenin in 3T3-L1 cells induced the expression of Slug, a transcription repressor, which subsequently inhibited TR4 binding to its cognate TR4 response element (TR4RE) in target gene promoters through a direct interaction with TR4. Slug-TR4 interaction led to suppression of TR4 transcriptional activity and lipid accumulation in 3T3-L1 adipocytes. Taken together, Wnt/β-catenin-Slug signaling inhibits adipogenesis of 3T3-L1 cells, in part, through repression of TR4 signaling.

## Materials and methods

### Cell culture, stable transfection, and differentiation

3T3-L1, NIH-3T3 and HEK293T cells were maintained in DMEM containing 10% newborn calf serum or 10% fetal bovine serum (Gibco, New York, USA). Stable cell populations were generated from a heterogeneous pool of Geneticin (Gibco, New York, USA) or Blasticidin (Invitrogen, Carlsbad, USA) resistant 3T3-L1 cells after transfection of pcDNA3, pcDNA3-Slug, pCI-neo, pCI-neo-β-catenin, pcDNA6.2-miR, or pcDNA6.2-SlugmiR. Adipocyte differentiation, Oil Red O staining, and quantitation of lipid accumulation were performed at least three times as described previously [1, 2].

### Plasmids and reporter gene assay

Plasmids pCMX-TR4 and pGL3-FATP1-DR1-Luc, and the various GST-TR4 constructs used in this study have been described previously [1, 19, 20]. Expression plasmid for full-length human β-catenin (pCI-neo-β-catenin) was provided by Dr. Bert Vogelstein (Johns Hopkins University, MD, USA) and expression plasmids for full-length mouse Snail (pcDNA3-mSnail) and Slug (pcDNA3-mSlug) were provided by Dr. Amparo Cano (Autonomous University of Madrid, Madrid, Spain) [21]. To generate pGEX4T-1-GST-Slug-2 construct, Slug-2 domain was amplified by PCR and cloned into pGEX4T-1 (GE Healthcare, Chicago, USA). Three copies of a consensus DR1 (cDR1) element and the DR1 region located in the pyruvate carboxylase (PC) promoter region were cloned into pGL3-luciferase (Promega, Madison, USA) to generate pGL3-cDR1-Luc and pGL3-PC-DR1-Luc. A pre-microRNA targeting *Slug* (Invitrogen, Carlsbad, USA) was cloned into pcDNA^TM^6.2-GW/EmGFP-miR expression vector (Invitrogen, Carlsbad, USA) to generate the pcDNA6.2-SlugmiR plasmid for silencing Slug. The vector with scrambled sequence (pcDNA6.2-miR) was used as a negative control. Transfection and reporter gene assays were performed as previously described [1]. Relative luciferase activity for each reporter gene was expressed as fold-induction relative to the empty plasmid (set as 1), and the results were expressed as mean as ± SD of three separate experiments.

### GST pull-down assay

GST pull-down assays were performed as described previously [20]. After the relative amount of purified GST-tagged protein was determined by Coomassie blue staining, equal amounts of GST- fused TR4 or Slug bound to glutathione-Sepharose-4B beads were incubated for 12 h at 4 °C with 5 μL of in vitro translated ^35^S-TR4 or ^35^S-Slug in 500 μL of GST binding buffer (50 mM HEPES, pH 7.6, 50 mM NaCl, 0.1% Nonidet P-40, 5 mM EDTA, and 10% glycerol). The beads were then washed three times with wash buffer (200 mM Tris–Cl pH 8.0, 500 mM NaCl, 0.1 mM EDTA, 0.1% Triton X-100, and 0.4 mM PMSF). The proteins were separated by 10% SDS-PAGE and visualized by autoradiography.

### Gel shift assay

Gel shift assays were performed as described previously [1, 22]. The following oligonucleotides were labeled with ^32^P and used for the gel shift assay: FATP1-DR1 (5′-AAGTGGGGCAAAGGGCACAGG-3′) and PC-DR1 (5′-GATCTCAATGTGACCCTTGCCCTCCATCA-3′).

### Chromatin immunoprecipitation (ChIP) assay

ChIP assays were performed in NIH-3T3 cells, as previously described [1, 23]. Samples were immunoprecipitated with normal IgG (Santa Cruz) and an anti-TR4 antibody (Santa Cruz) at 4 °C overnight. ChIP assays were performed in NIH-3T3 cells using normal IgG (Santa Cruz, Dallas, USA) and an anti-TR4 antibody (Santa Cruz, Dallas, USA), as previously described [1]. The following primers were used for PCR detection of a mouse FATP1 promoter that contains a TR4RE: 5ʹ-CCCTCGAGGCACTCTATAAACTAGGAG-3ʹ and antisense, 5 ʹ-ACAGGTAAAGACACTGAATG-3ʹ. The following primers were used for PCR detection of a mouse FATP1 promoter region lacking a TR4RE: sense, 5ʹ-TTCTTCCCAAGGAGAACTGT-3ʹ and antisense, 5 ʹ-CCAAGCTTTTCTCACAGAAGTCTGGACT-3ʹ.

### Immunoblotting

Immunoblotting was performed as described previously [1]. The proteins were probed with an anti-CD36 (Abcam, Cambridge, UK), anti-FATP1 (biorbyt, Cambridge, UK), anti-β-catenin, anti-aP2 (Cell Signaling, Danvers, USA), anti-PC, anti-β-actin, anti-TR4, or anti-Slug (Santa Cruz, Dallas, USA) antibody, followed by probing with a horseradish peroxidase-conjugated secondary antibody (Santa Cruz, Dallas, USA) or alkaline phosphatase-conjugated secondary antibody (Santa Cruz, Dallas, USA) [1, 24].

### Real-time quantitative RT-PCR (RT-qPCR)

RT-qPCR was performed using a Corbett Rotor-Gene 6000 (Qiagen, Hilden, Germany), and the relative abundance of the target mRNA was quantified relative to 36B4 expression. The sequences of *Tr4*, *Fatp1*, *Pc*, and *36b4* primers used for RT-qPCR have been reported previously [1, 2]. The sequences of the primers used for other genes were as follows: β-catenin: sense, 5ʹ-TGGACAATGGCTACTCAAG-3ʹ and antisense, 5ʹ-GTCAACATCTTCTTCCTCAG-3ʹ; *Pparγ*: sense, 5ʹ- TGCTGTTATGGGTGAAACTCTGGG-3ʹ and antisense, 5ʹ- CGCTTGATGTCAAAGGAA TGCG-3ʹ; *Ap2*: sense, 5ʹ-ACACCGAGATTTCCTTCAAACTG-3ʹ and antisense, 5ʹ- CCATCTAGGG TTATGATGCTCTTCA-3ʹ; Slug: sense, 5ʹ-TCGCCTGGACCGTTATCTG-3ʹ and antisense, 5ʹ-CGCTGTAGTTGGGCTTCTTG-3ʹ; and Snail: sense, 5ʹ-GGATGTGAAGAGATACCAGTG-3ʹ and antisense, 5′-AAGATGCCAGCGAGGATG-3ʹ. Each group was analyzed in triplicate using total RNA from three separate experiments.

### Statistical analysis

The statistical analysis of data was performed by the student’s *t*-test. The data are expressed as means ± SD. All the experiments were performed at least in triplicate.

## Results

### TR4 transcriptional activity is negatively regulated by β-catenin

**Fig. 1:**
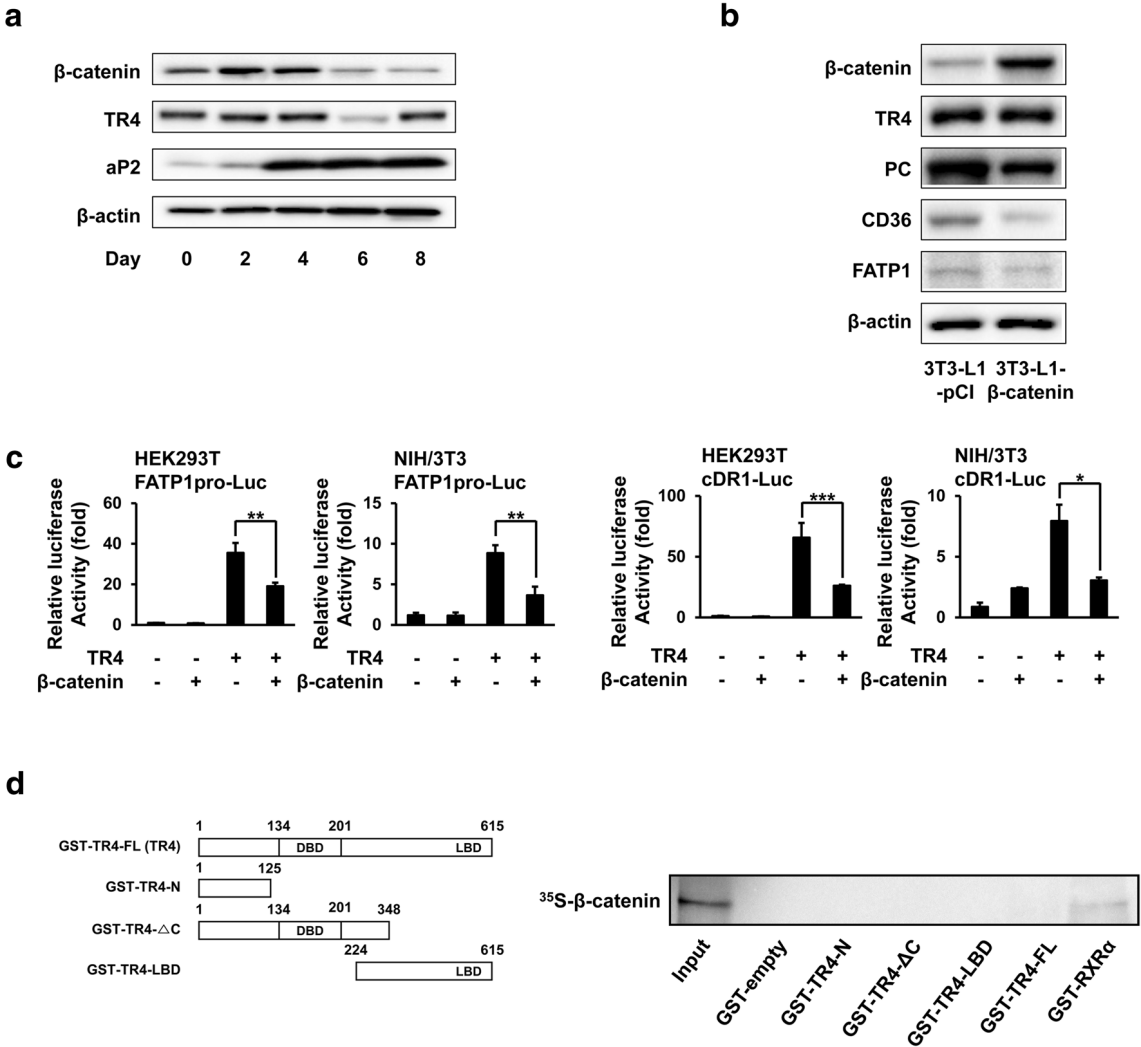
β-catenin inhibits TR4 target gene expression by repressingTR4 transcriptional activity. **a** Immunoblots of β-catenin, TR4, aP2, and β-actin during 3T3-L1 adipogenesis. **b** Effect of β-catenin on the expression of TR4 target genes. Protein levels of β-catenin, TR4, PC, FATP1, CD36, and β-actin were analyzed by immunoblotting in β-catenin-overexpressing (3T3-L1-β-catenin) or control 3T3-L1 (3T3-L1-pCI) adipocytes at day 5. **c** Luciferase assays in HEK293T or NIH-3T3 cells after transfection of reporter plasmids along with TR4 and β-catenin expression plasmids, as indicated. (*p < 0.01, **p < 0.005, and ***p < 0.001 vs. control) **d** Interaction between TR4 and β-catenin was analyzed with a GST pull-down assay. The different GST-TR4 constructs used were described previously [24] and are illustrated schematically on the upper panel. Input: 20% of the labeled protein used in the GST pull-down assay

Previous reports showed that Wnt/β-catenin signaling suppresses the differentiation of 3T3-L1 preadipocytes and that β-catenin levels decrease as adipogenesis progresses [25]. To determine the correlation between TR4 and β-catenin during adipogenesis, we compared their relative protein levels during the differentiation of 3T3-L1 preadipocytes using immunoblotting. Consistent with previous reports, β-catenin was highly expressed in 3T3-L1 preadipocytes and then gradually decreased as adipocyte differentiation progressed (Fig. 1a). In contrast, TR4 levels were relatively constant and the levels of aP2, an adipogenic marker, were dramatically increased during this period. To further examine the possible role of β-catenin in the regulation of TR4 activity, we next generated pools of stable 3T3-L1 preadipocytes constitutively expressing β-catenin (3T3-L1-β-catenin), and determined the effect of β-catenin overexpression on the expression of TR4 and TR4 target genes. The protein levels of the TR4 target genes, Fatp1, and Cd36, on day 5 of differentiation, were dramatically reduced by 56% and 67%, respectively, due to β-catenin overexpression (Fig. 1b). Next, we performed a reporter gene assay to determine whether β-catenin suppresses TR4 transcriptional activity. As expected, TR4 dramatically induced the activity of the luciferase reporter gene fused to the mouse FATP1 promoter (FATP1pro-Luc) in both HEK293T and NIH-3T3 cells (Fig. 1c). However, when a β-catenin expression plasmid was co-transfected with TR4, TR4 transcriptional activation of FATP1pro-Luc was reduced by ~ 53% and 60%. In addition, β-catenin also suppressed TR4-mediated induction of luciferase activity from the cDR1-Luc construct, which contains three copies of a consensus DR1 sequence, an ideal TR4RE. β-catenin is known to regulate the expression of target genes through interaction with transcription factors such as TCF4 and NF-κB [26, 27]. To determine whether β-catenin inhibits TR4 activity via a physical interaction with TR4, we performed a GST pull-down assay. As shown in Fig. 1d, while ^35^S-labeled β-catenin interacted with GST-RXRα (a positive control), it was not able to interact with either the full-length GST-TR4 fusion protein (GST-TR4-FL) or the different deletion mutants of TR4 fused to GST (GST-TR4-N [aa 1–125], GST-TR4-∆C [aa 1–348], and GST-TR4-LBD [aa 224–615]).

### β-catenin inhibits TR4 target gene expression through induction of Slug expression

**Fig. 2:**
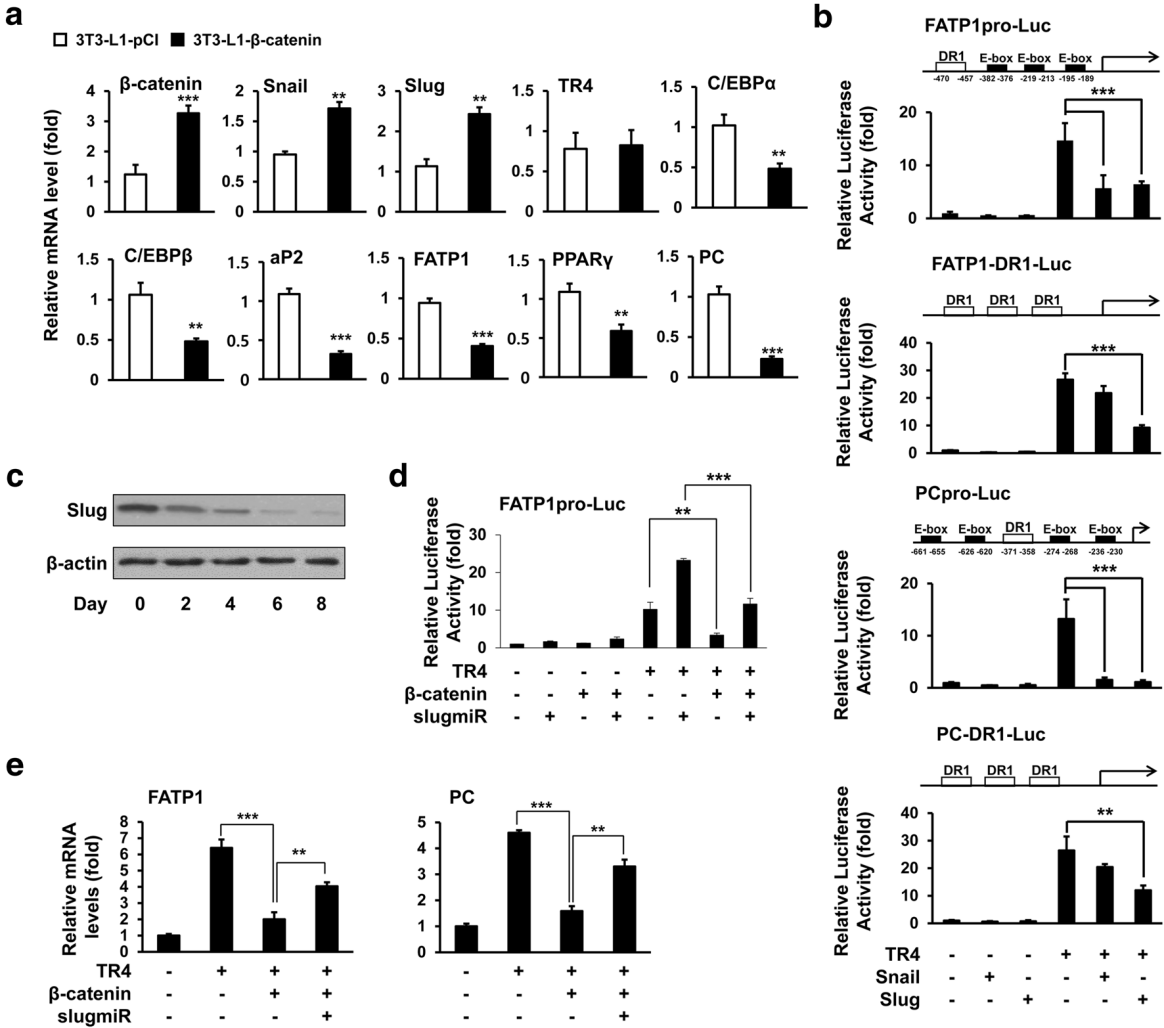
Slug is required for β-catenin-mediated suppression of TR4 activity. **a** RT-qPCR analyses of gene expression in day 5 3T3-L1-β-catenin or 3T3-L1-pCI adipocytes. **b** and **d**. Luciferase assays in the HEK293T (FATP1-DR1-Luc and PC-DR1-Luc) and NIH-3T3 (FATP1pro-Luc and PCpro-Luc) cells after transfection of reporter plasmids along with TR4, Snail, Slug, SlugmiR, or β-catenin expression plasmids, as indicated. **c** Immunoblots of Slug and β-actin during 3T3-L1 adipogenesis. **e** RT-qPCR analyses of *Fatp1* and *Pc* mRNA levels in 3T3-L1-C and 3T3-L1-TR4 adipocytes at day 8 after 48 h transfection with SlugmiR or β-catenin expression plasmids as indicated. (**p < 0.005, and ***p < 0.001)

Wnt/β-catenin signaling plays its role, in part, via induction of various transcription factors. Accumulating evidence shows that Snail and Slug can be induced by β-catenin and mediate the effects of β-catenin during a variety of cellular events, including epithelial-mesenchymal transition and cell proliferation [16, 28]. Thus, β-catenin may exert its inhibitory effect against TR4 function and adipogenesis of 3T3-L1 preadipocytes by inducing these genes. To test this hypothesis, we first determined whether β-catenin could induce Snail and Slug expression in 3T3-L1 preadipocytes. As shown in Fig. 2a, on day 5 of differentiation, the mRNA levels of *Snail* and *Slug* in 3T3-L1-β-catenin adipocytes were 1.8- and 2.4-fold higher, respectively, compared to control adipocytes (3T3-L1-pCI) along with decreased expression of TR4 target genes, *Fatp1* (58%) and *Pc* (79%). We next determined the effects of Snail and Slug on TR4 transcriptional activity in HEK293T cells using a reporter assay. As expected, TR4 strongly increased the promoter activity of FATP1 (FATP1pro-Luc) and PC (PCpro-Luc) (Fig. 2b). However, TR4-mediated transcriptional activation of these reporter genes was dramatically repressed when a Snail- or Slug-expressing plasmid was co-transfected with TR4 expression plasmid into HEK293T cells. Interestingly, we found E-box sites that are located close to the TR4REs in both the FATP1 and PC promoters. Thus, to determine whether the inhibitory effects of these Snail family genes on TR4 transcriptional activity is due to the presence of E-boxes, Snail family binding sites, in FATP1 and PC promoters, we generated two different reporter genes containing the three TR4REs from each promoter without E-box. The results of the reporter gene assay showed that Slug, but not Snail, suppressed TR4-mediated transactivation of FATP1-DR1-Luc and PC-DR1-Luc (Fig. 2b).

Previous studies have reported that unlike the flexible binding capacity of Snail to various E box sequences, Slug binds to the E box only in the presence of “GG” or “CC” in the E box (CANNTG) [29]. Interestingly, sequence analysis revealed that all the E boxes in the FATP1 and PC promoters do not have “CAGGTG” or “CACCTG” sequences [1, 2]. Previous studies and our data suggest that Slug inhibits TR4 transactivation in an E-box-independent manner. Therefore, we decided to determine how Slug negatively regulates TR4 activity. We first examined the expression profile of Slug during adipocyte differentiation using immunoblotting. Although, Slug was highly expressed in 3T3-L1 preadipocytes, its expression was dramatically decreased at day 2 and almost disappeared after 6 days of differentiation (Fig. 2c), indicating that Slug and β-catenin levels decreased together during adipocyte differentiation. We next determined whether Slug knockdown could abolish β-catenin-induced suppression of TR4 transcriptional activity using a reporter gene assay. TR4 induced FATP1 promoter activity in NIH-3T3 cells (Fig. 2d). Moreover, Slug knockdown with a Slug-specific microRNA (SlugmiR) significantly enhanced TR4-mediated induction of FATP1 promoter activity (approximately 2.5-fold) compared to the cells transfected with control microRNA. As expected, when β-catenin was cotransfected with TR4, TR4 transactivation was reduced by approximately 75%. However, β-catenin-mediated inhibition of TR4 activity was completely abolished by the addition of SlugmiR. RT-qPCR analysis revealed that, on day 8 of differentiation, the mRNA levels of both *Fatp1* and *Pc* in 3T3-L1 adipocytes overexpressing TR4 (3T3-L1-TR4) were upregulated compared with control adipocytes (3T3-L1-C) (Fig. 2e). In contrast, overexpression of β-catenin strongly suppressed TR4-mediated induction of *Fatp1* and *Pc* expression in 3T3-L1-TR4 adipocytes. However, when SlugmiR was cotransfected with β-catenin, suppressive effect of β-catenin was strongly inhibited. These results showed that Slug mediates the inhibitory effect of β-catenin on transcriptional activity.

### Slug suppresses lipid accumulation in 3T3-L1 adipocytes

**Fig. 3:**
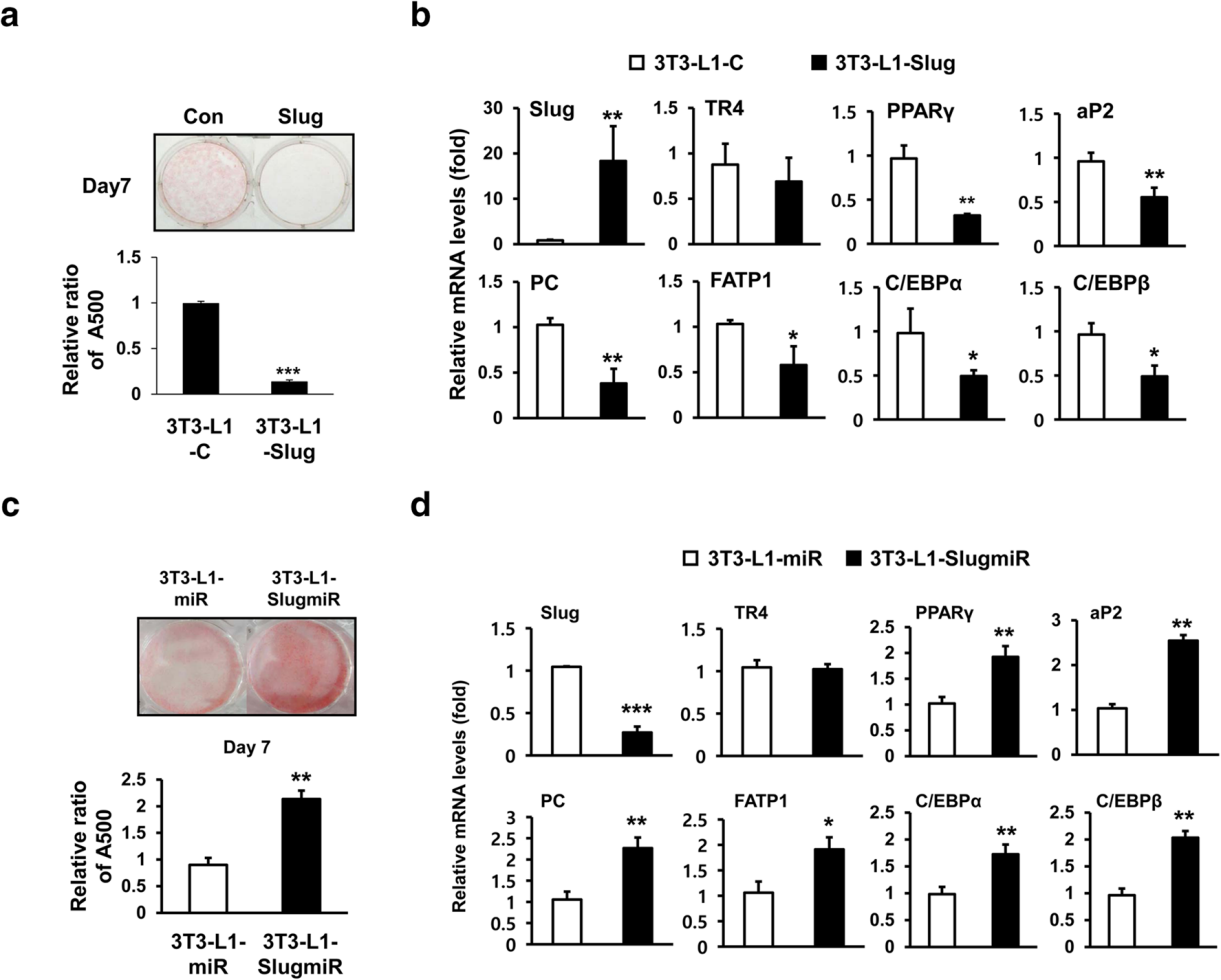
Effect of Slug on lipid accumulation and lipogenic gene expression in 3T3-L1 adipocytes. **a** and **c** 3T3-L1 adipocytes (3T3-L1-Slug, 3T3-L1-C, 3T3-L1-SlugmiR, and 3T3-L1-miR) were stained with Oil Red O on the indicated days after induction of differentiation. **b** and **d** The mRNA levels of different genes were quantitated by RT-qPCR in 3T3-L1 adipocytes (3T3-L1-Slug, 3T3-L1-C, 3T3-L1-SlugmiR, or 3T3-L1-miR) on day 9 after induction of differentiation. (*p < 0.01, **p < 0.005, and ***p < 0.001 vs. control)

To further determine the role of Slug in β-catenin-induced suppression of TR4 function and adipogenesis, we next generated pools of 3T3-L1 cells stably overexpressing Slug (3T3-L1-Slug) and analyzed the effect of Slug on adipogenesis in 3T3-L1 preadipocytes using Oil Red O staining. As shown in Fig. 3a, lipid accumulation was significantly inhibited in day 7 3T3-L1-Slug adipocytes compared to the levels in control adipocytes stably transfected with an empty vector (3T3-L1-C). Furthermore, in day 9 3T3-L1-Slug adipocytes, the mRNA levels of two TR4 target genes *Fatp1* and *Pc* were 40% and 70% lower, respectively, than those in control adipocytes (3T3-L1-C) (Fig. 3b). In contrast, silencing of Slug in 3T3-L1 preadipocytes, by constitutively expressing SlugmiR (3T3-L1-SlugmiR) enhanced lipid accumulation and mRNA levels of lipogenic genes (*Fatp1* and *Pc*) in 3T3-L1 adipocytes on day 7 and day 9, respectively, as compared to those of control 3T3-L1 adipocytes (Fig. 3c and d). As expected, TR4 mRNA levels in 3T3-L1 adipocytes were not affected by Slug knockdown. These results suggest that Slug strongly inhibits adipogenesis of 3T3-L1 preadipocytes.

### Slug inhibits TR4 transcriptional activity via physical interaction with TR4

**Fig. 4:**
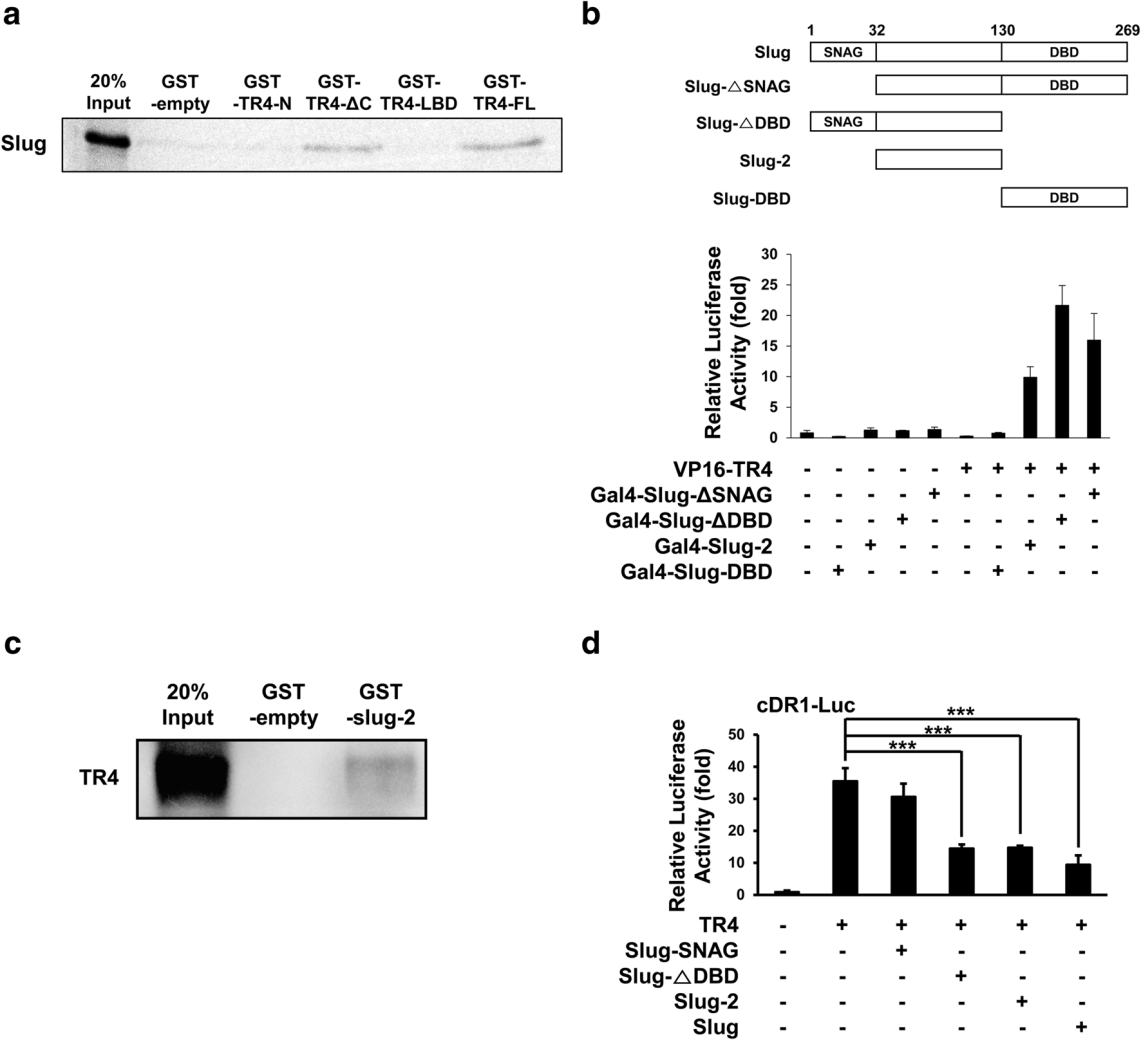
Slug downregulates TR4 transcriptional activity via direct interaction with TR4. **a**, **c**. Interaction between TR4 and Slug was analyzed using GST pull-down assay. Equal amounts of in vitro translated ^35^S-labled Slug or ^35^S-labled TR4 were incubated with purified GST fusion proteins bound to glutathione-Sepharose beads, as indicated. **b** A mammalian two-hybrid assay was performed in HEK293T cells by co-transfection of the pG5-Luc plasmids with different Gal4-Slug constructs and VP16-TR4, as indicated. The different Gal4-Slug constructs used for this assay are illustrated schematically on the upper panel. **d** A reporter plasmid was co-transfected into HEK293T cells along with TR4 expression plasmids and different Slug fragments, as indicated. (***p < 0.001)

To address the molecular mechanism by which Slug suppresses TR4 function in lipogenesis, we tested whether Slug physically interacts with TR4. First, we performed a GST pull-down assay using various deletion mutants of TR4 fused to GST. Only GST-TR4-FL and GST-TR4-ΔC were able to interact with Slug (Fig. 4a). Next, we performed a mammalian two-hybrid assay to determine which region of Slug is required for its interaction with TR4. When VP16-TR4 was co-transfected with either GAL4-Slug-ΔDBD (aa 1–130) or GAL4-Slug-ΔSNAG (aa 33–269), but not with GAL4-Slug-ΔDBD (aa 131–269), strong pG5-Luc activity was observed in HEK293T cells (Fig. 4b). In addition, GAL4-Slug-2 (aa 33–130), which contains a region of Slug present in both GAL4-Slug-ΔDBD and GAL4-Slug-ΔSNAG, also showed a strong interaction with VP16-TR4. Furthermore, Slug-2 fused to GST (GST-Slug-2), but not GST alone, interacted with TR4, indicating that the Slug-2 domain is responsible for Slug binding to TR4 (Fig. 4c). To further confirm whether the Slug-2 region is sufficient to repress TR4 transactivation, we performed a reporter gene assay in HEK293T cells using cDR1-Luc. As expected, TR4 significantly induced cDR1-Luc activity, and this TR4 transactivation was dramatically repressed not only by full-length Slug, but also by Slug-2 (Fig. 4d). In contrast, the SNAG region (Slug-SNAG) did not affect TR4 transcriptional activity.

### Slug suppresses TR4-enhanced lipid accumulation in 3T3-L1 adipocytes by interrupting TR4 homodimerization

**Fig. 5:**
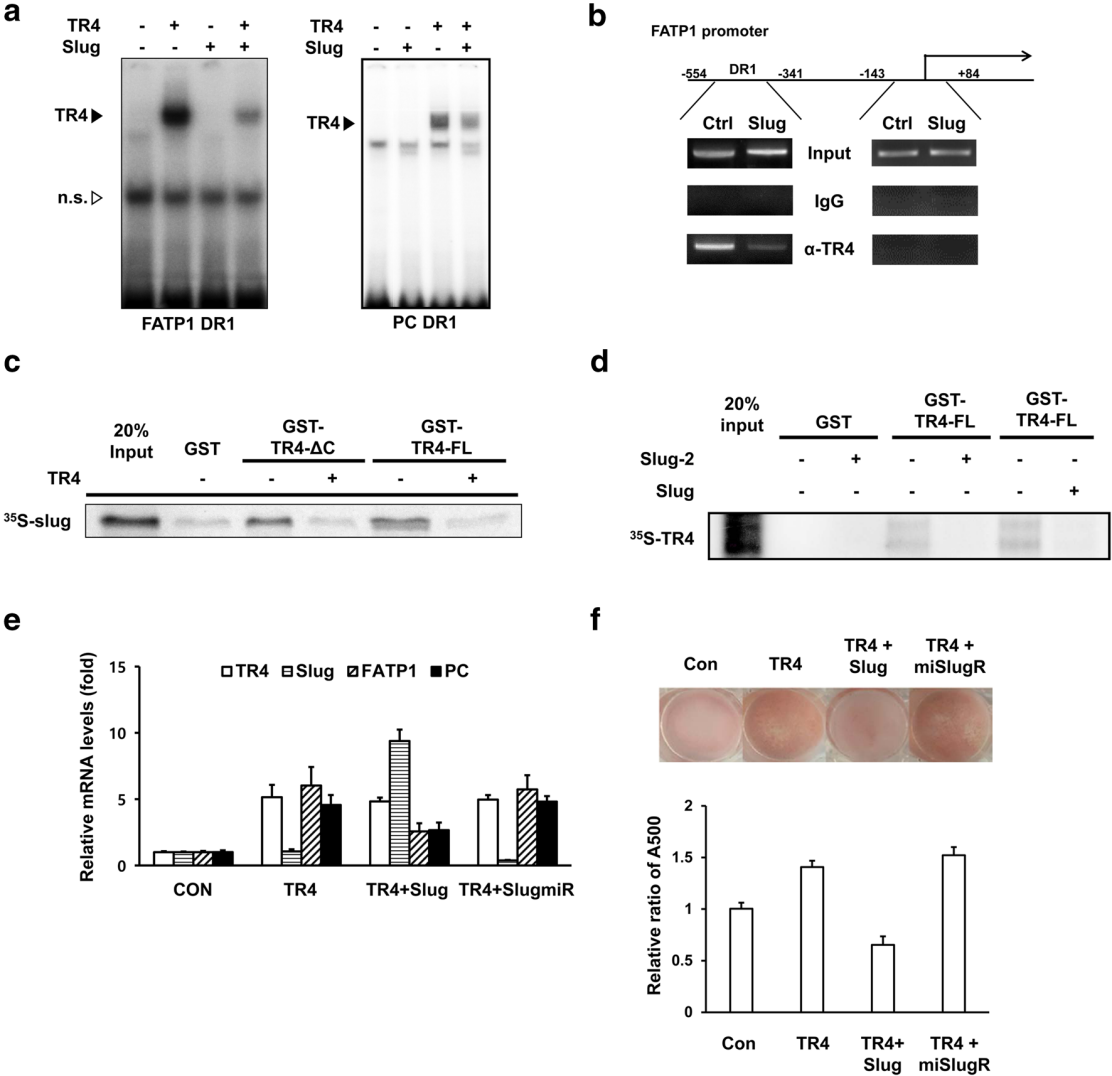
Slug inhibits TR4-TR4RE interaction and TR4 function by suppressing TR4 homodimer formation. **a** Gel shift assays using ^32^P-labeled FATP1-DR1 or PC-DR1. Mock lysate, in vitro translated TR4, and Slug were incubated with ^32^P-labeled FATP1-DR1 or PC-DR1, as indicated. **b** A ChIP assay in 3T3-L1-Slug and 3T3-L1-C cells using an anti-TR4 antibody or normal IgG. The region (− 554 bp to − 341 bp) containing a TR4RE and an E box, and the 3ʹ downstream region (− 143 bp to + 84 bp) lacking both, a TR4RE and an E box of the FATP1 promoter were amplified with immunoprecipitates using PCR. **c** The effect of TR4 on the interaction between ^35^S-labeled Slug and purified GST-TR4 protein (GST-TR4-∆C or GST-TR4-FL) was analyzed by a GST pull-down assay. **d** The effect of Slug on TR4 homodimer formation. In vitro translated ^35^S-labeled TR4 was incubated with either GST or GST-TR4-FL in the absence or presence of in vitro translated Slug and Slug-2. **e** and **f** After 48 h transfection of Slug expression plasmids or SlugmiR plasmids in adipocytes (3T3-L1-TR4 or 3T3-L1-C), mRNA levels of indicated genes (**e**) and lipid accumulation (**f**) were determined at day 8 by RT-qPCR and Oil Red O staining, respectively

Given that the inhibitory role of Slug against TR4 activity is mediated through its physical interaction with TR4, Slug may inhibit TR4 transcriptional activity by preventing TR4 from binding to its cognate response elements. To prove this hypothesis, we performed a gel shift assay using TR4RE sequences located in the TR4 target gene promoters (FATP1-DR1 and PC-DR1) as probes. As shown in Fig. 5a, TR4, but not Slug, formed complexes with both DR1 sequences, indicating that Slug-mediated inhibition of TR4 activity is not due to competition with TR4 for binding to TR4REs. However, these TR4-DNA complexes were dramatically reduced in the presence of Slug. Next, we prepared chromatin samples from 3T3-L1-C and 3T3-L1-Slug cells, and then, performed a ChIP assay using an anti-TR4 antibody. PCR analysis showed that recruitment of TR4 to the FATP1 promoter region (− 554 bp to − 341 bp) containing FATP1-TR4RE and an E box, but not to the downstream region (− 143 bp to + 84 bp) lacking a TR4RE and an E box, was observed in chromatin samples prepared from both control and Slug-overexpressing cells. However, this PCR amplification was markedly lower in Slug-overexpressing cells than in control cells (Fig. 5b). Because TR4 usually binds to TR4REs as a homodimer and Slug inhibits TR4 binding to TR4REs, we tested whether Slug affects the formation of TR4-TR4 homodimer. As shown in Fig. 5c, ^35^S-labeled Slug could be pulled down by both GST-TR4-ΔC and GST-TR4-FL in the absence of TR4. However, when ^35^S-Slug was incubated with GST-TR4-ΔC or GST-TR4-FL in the presence of non-radiolabeled TR4, no interaction between Slug and either GST-TR4-ΔC or GST-TR4-FL was observed. Furthermore, non-radiolabeled Slug or Slug-2 also inhibited the binding between GST-TR4-FL and ^35^S-labeled TR4, indicating that Slug suppresses TR4 binding to TR4REs through inhibition of TR4 homodimerization (Fig. 5d). We next determined whether Slug can suppress TR4-mediated induction of target genes in 3T3-L1 adipocytes stably overexpressing TR4 (3T3-L1-TR4) using RT-qPCR analysis. On day 8 of differentiation, mRNA levels of both *Fatp1* and *Pc* in 3T3-L1-TR4 adipocytes were upregulated by 4.8-fold, as compared with those in control adipocytes (Fig. 5e). However, when Slug was introduced into 3T3-L1-TR4 adipocytes, the mRNA levels of these genes were significantly reduced with no change in TR4 mRNA levels (Fig. 5e). In contrast, when Slug was silenced in day 6 3T3-L1-TR4 adipocytes by transfection of SlugmiR, mRNA levels of *Fatp1* and *Pc* were slightly increased as compared with those in 3T3-L1-TR4 adipocytes. We next determined whether Slug affects TR4-mediated lipid accumulation in 3T3-L1 adipocytes. As expected, TR4 enhanced lipid accumulation in day 8 3T3-L1 adipocytes by 44%, as compared with that in control adipocytes (Fig. 5f). When Slug was overexpressed in day 8 3T3-L1-TR4 adipocytes, TR4-induced lipid accumulation was reduced by 39%. However, Slug knockdown by SlugmiR did not significantly affect TR4-mediated lipid accumulation in day 8 3T3-L1-TR4 adipocytes. Together, these results show that Slug exhibits inhibitory effect on adipogenesis, at least in part, via inhibition of TR4 activity.

## Discussion

In this study, we demonstrated that β-catenin negatively regulates TR4 transcriptional activity by inducing Slug, which, in turn, displaces TR4 from its cognate DNA binding sites located in promoter regions of its target genes via a direct interaction with TR4 in 3T3-L adipocytes. β-catenin is a key mediator of the Wnt signaling pathway, and the inhibitory role of the Wnt/β-catenin signaling pathway during adipogenesis in 3T3-L1 preadipocytes has been extensively studied. Accordingly, overexpression of β-catenin in 3T3-L1 preadipocytes blocked adipogenesis and concomitantly reduced the expression of TR4 target genes without affecting the levels of TR4. However, although β-catenin suppresses TR4 transcriptional activity, no physical interaction between β-catenin and TR4 was detected in this study. Growing evidence suggests that Wnt/β-catenin signaling regulates various transcriptional pathways in many cells by altering either expression or activity of downstream transcription regulators, such as members of the Snail family. Consistent with previous reports [14, 15], overexpression of β-catenin in 3T3-L1 preadipocytes resulted in increased expressions of Snail and Slug, two members of the Snail family (Fig. 2a). Snail and Slug have been shown to negatively regulate gene expression by binding to the E-box in target gene promoters [30, 31]. However, when we used reporter genes linked to FATP1-DR1 or PC-DR1 constructs that do not include any E-boxes, Slug, but not Snail, negatively regulated TR4 transcriptional activity. Consistent with these results, Slug suppressed the expression of TR4 target genes in 3T3-L1 adipocytes along with reduction of lipid accumulation, while its silencing enhanced TR4 target gene expression and lipid accumulation in 3T3-L1 adipocytes. Furthermore, Slug overexpression in 3T3-L1-TR4 adipocytes repressed the expression of TR4 target genes such as *Fatp1* and *Pc*, and led to a dramatic decrease in lipid accumulation when adipogenic stimuli were applied, suggesting that Slug might inhibit lipid accumulation in mature adipocytes by suppressing TR4 activity. Interestingly, Slug knockdown in day 8 3T3-L1-TR4 adipocytes using SlugmiR did not significantly affect lipogenic function of TR4. Since Slug levels are already very low in the mature 3T3-L1 adipocytes as shown in Fig. 2c, and TR4 is overexpressed in 3T3-L1-TR4 adipocytes, TR4 may perform its lipogenic role in the adipocytes regardless of additional decrease in Slug levels.

TR4 and Slug often cross-talk with other signaling pathways via physical interaction with other signaling molecules [20, 32, 33]. Indeed, we observed that Slug directly interacts with TR4 and disrupts the formation of TR4 homodimers, thereby displacing TR4 from the TR4REs located in its target gene promoters, which may lead to suppression of TR4-mediated adipogenesis in 3T3-L1 preadipocytes. In contrast to 3T3-L1 preadipocytes cell line, Slug overexpression increased the size of white adipose tissue in mice, and mouse embryonic fibroblasts derived from these mice showed enhanced adipogenic potential [34]. Several studies have reported that the results obtained from cell line models are different from those obtained from in vivo systems [11, 35], suggesting that this may reflect the difference between a biological characteristic of 3T3-L1 cell line and in vivo models, although, 3T3-L1 preadipocyte is a well characterized model for the study of adipogenesis.

Chronic overnutrition leads to dysregulation of lipid metabolism, such as lipogenesis and fatty acid oxidation. The dysregulation, in combination with the increased flux of dietary fatty acids, promotes excessive fat accumulation in the adipose tissue, resulting in obesity and associated metabolic disorders including diabetes, fatty liver diseases, and cardiovascular diseases [36–38].

TR4 is a key transcriptional regulator of adipocyte lipid metabolism, implying that dysregulation of TR4 activity might contribute to metabolic diseases. Consistent TR4 deficiency in mice showed reduced adiposity and increased insulin sensitivity, suggesting the clinical importance of TR4 [4]. Recent studies showing TR4 activation by thiazolidinediones, an antidiabetic drug, further supports the importance of TR4 in the development of metabolic disorders [39]. Similar to other nuclear receptors, TR4 could be regulated by small molecules or protein modulators and has a role in energy metabolism. Hence, identification of a TR4 modulator such as Slug might facilitate understanding of the role of TR4 in the incidence and progression of various metabolic disorders, including diabetes, cardiovascular diseases, and metabolic cancers.

Considered together, our findings suggest that Slug mediates the β-catenin-induced inhibition of TR4 transcriptional activity by suppressing the TR4 DNA binding activity, which in turn suppresses the 3T3-L1 adipogenesis.

## Data Availability

The data used to support the findings of this study are available from the corresponding author upon request.

## Abbreviations

cDR1: Consensus direct repeat 1
C/EBP: CCAAT-enhancer binding protein
ChIP: Chromatin Immunoprecipitation
FATP1: Fatty acid transport protein 1
GST: Glutathione-S-transferase
PC: Pyruvate carboxylase
PPAR: Peroxisome proliferator-activated receptor
TR4: Testicular receptor 4
TR4RE: TR4 response element
